# Supplementary figures and images for: Natural Variation Uncovers Candidate Genes for Barley Spikelet Number and Grain Yield under Drought Stress
Source: Genes (Basel). 2020 May 11;11(5):533. doi: 10.3390/genes11050533 (PMC7290517; doi:10.3390/genes11050533)

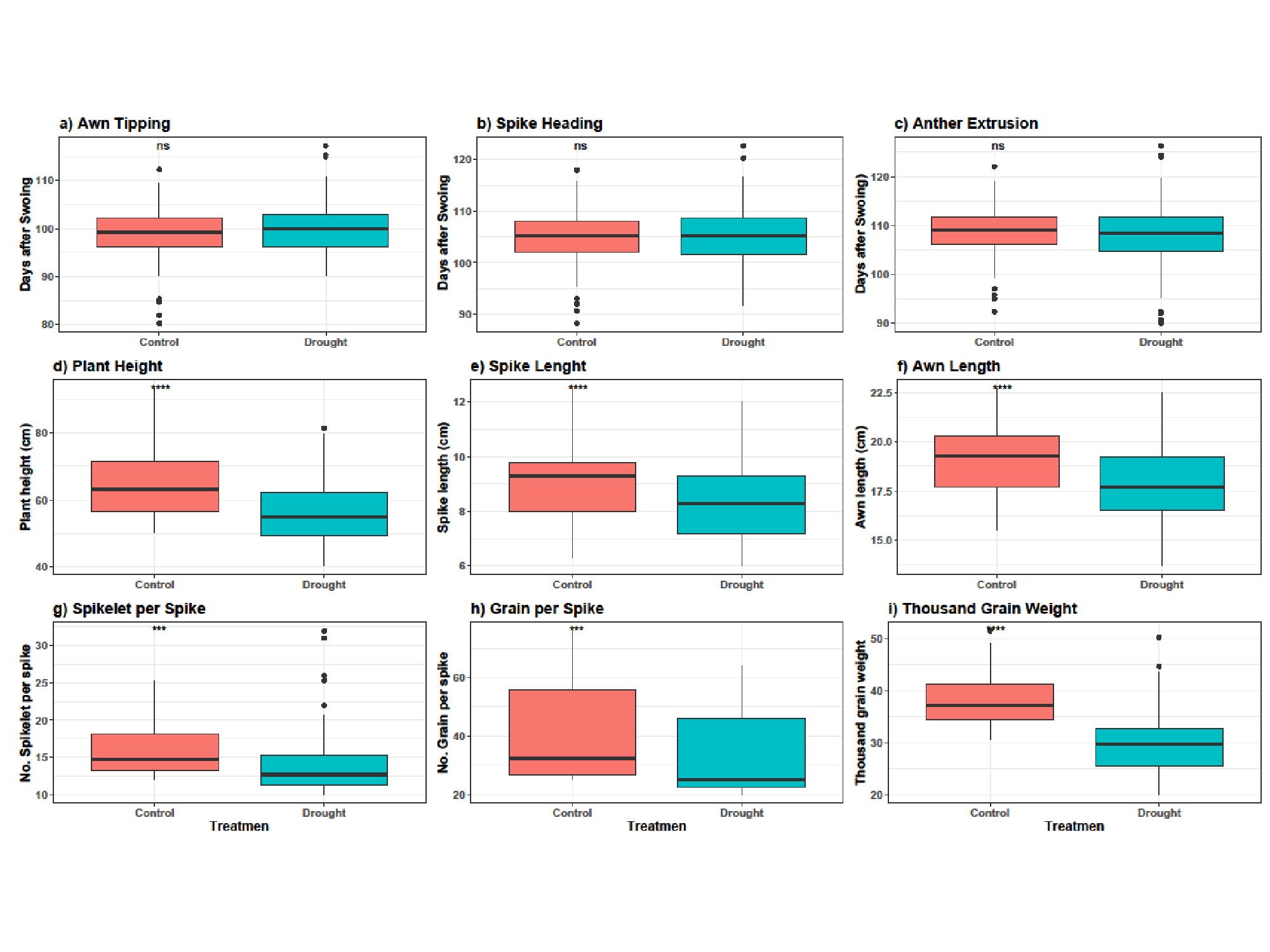

Supplement: Supplementary file 1 [file genes-11-00533-s001.zip › genes-765865-supplementary/Figure S3.jpg]

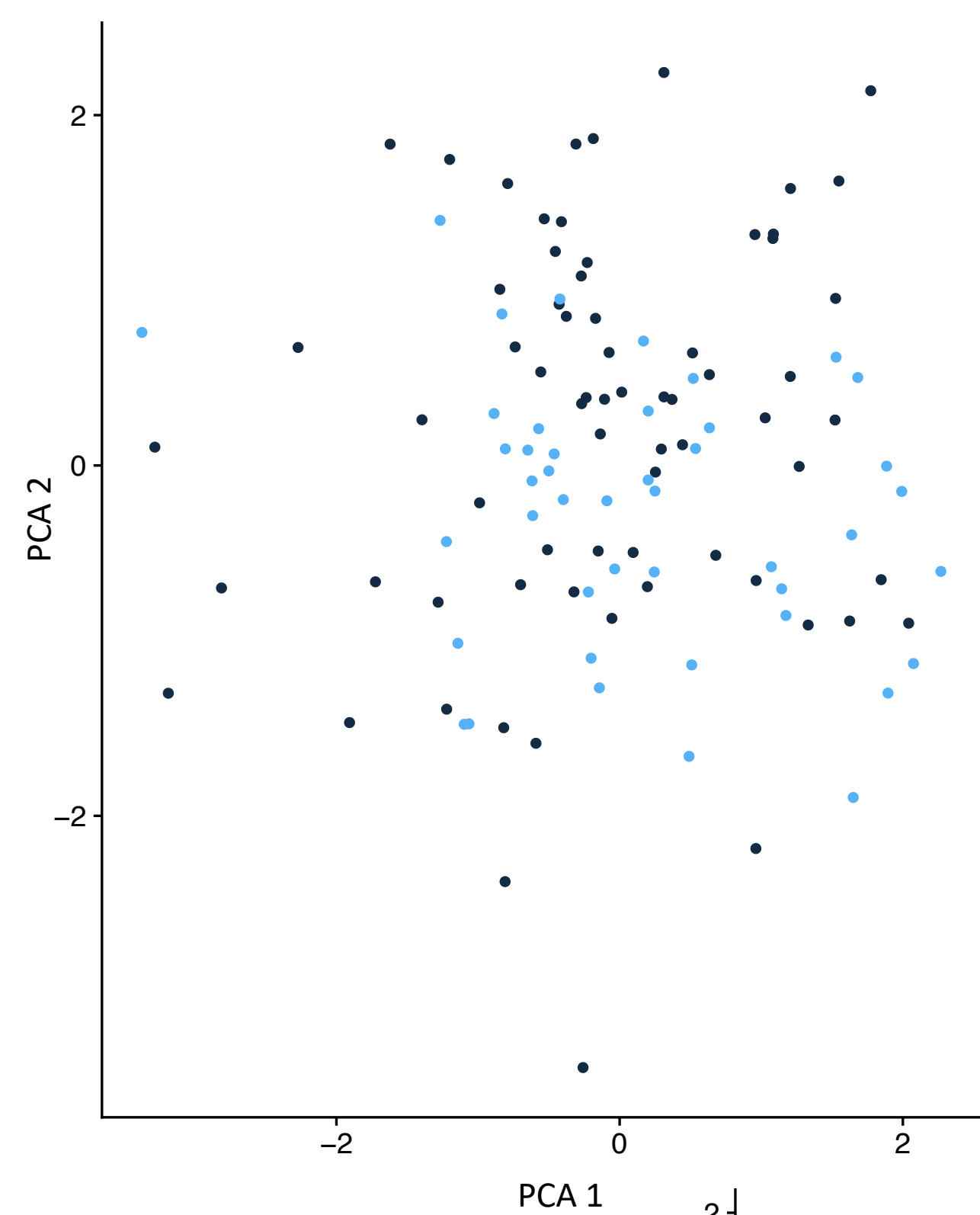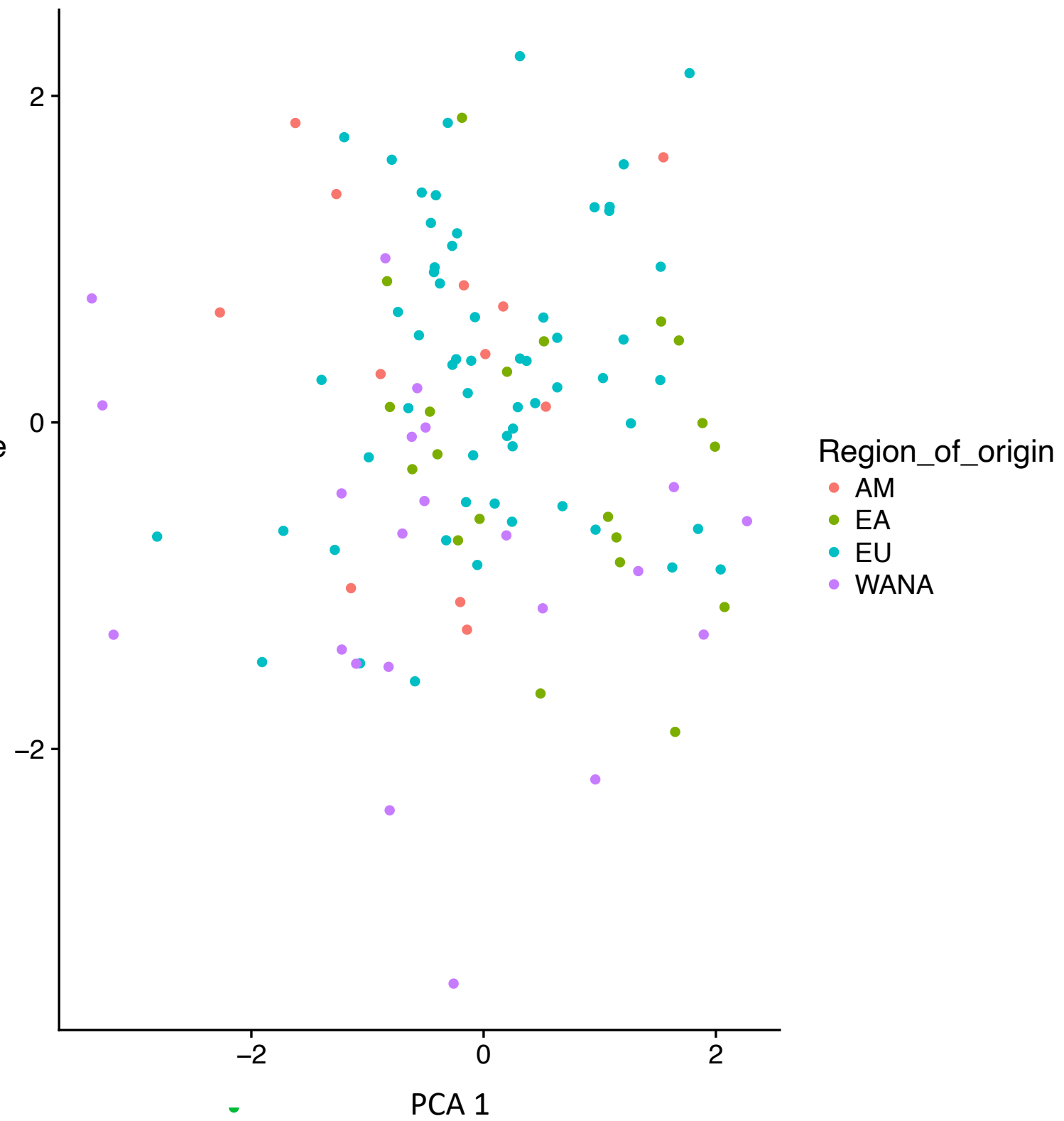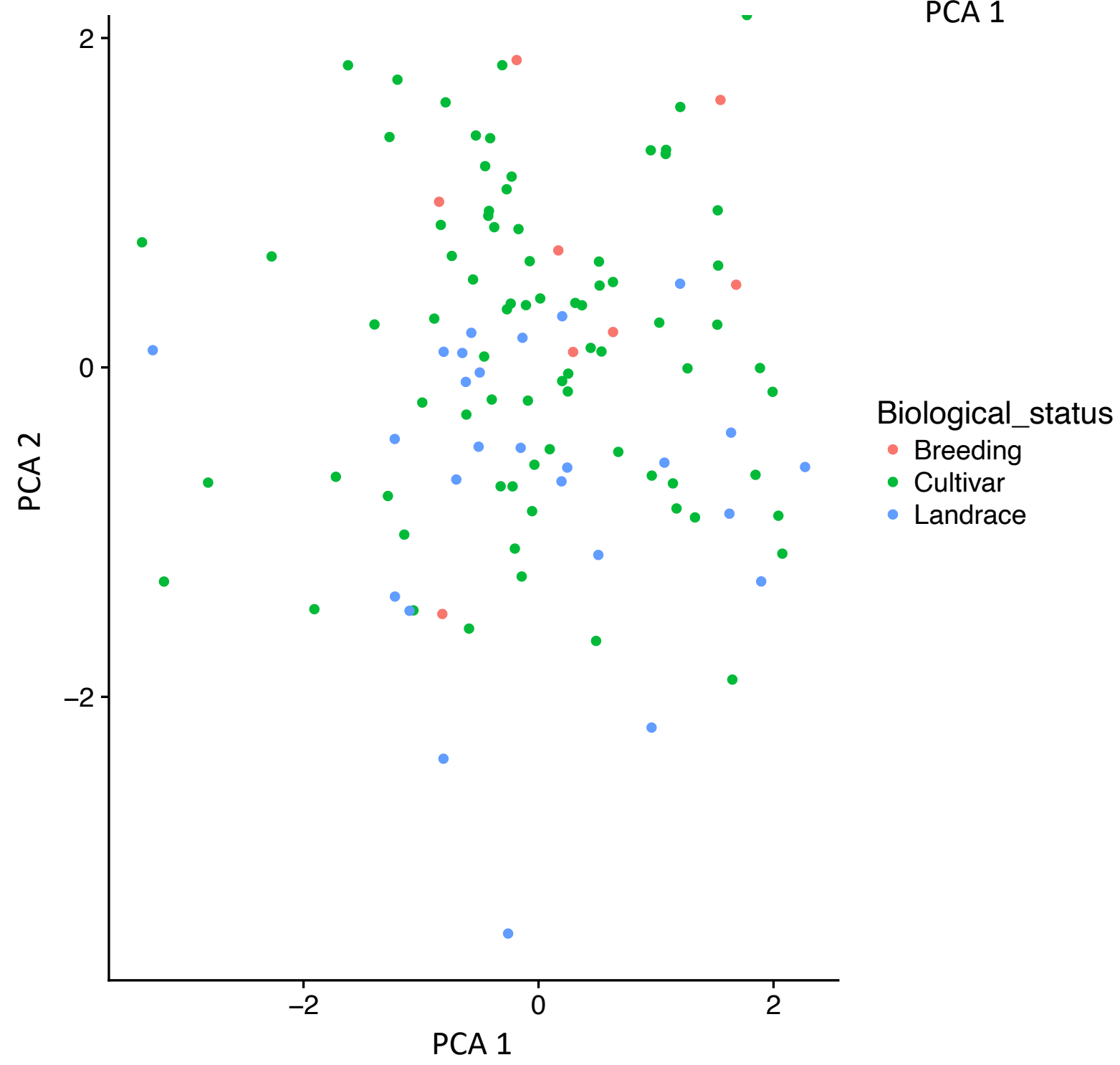

Supplement: Supplementary file 1 [file genes-11-00533-s001.zip › genes-765865-supplementary/Figure S1.pdf]

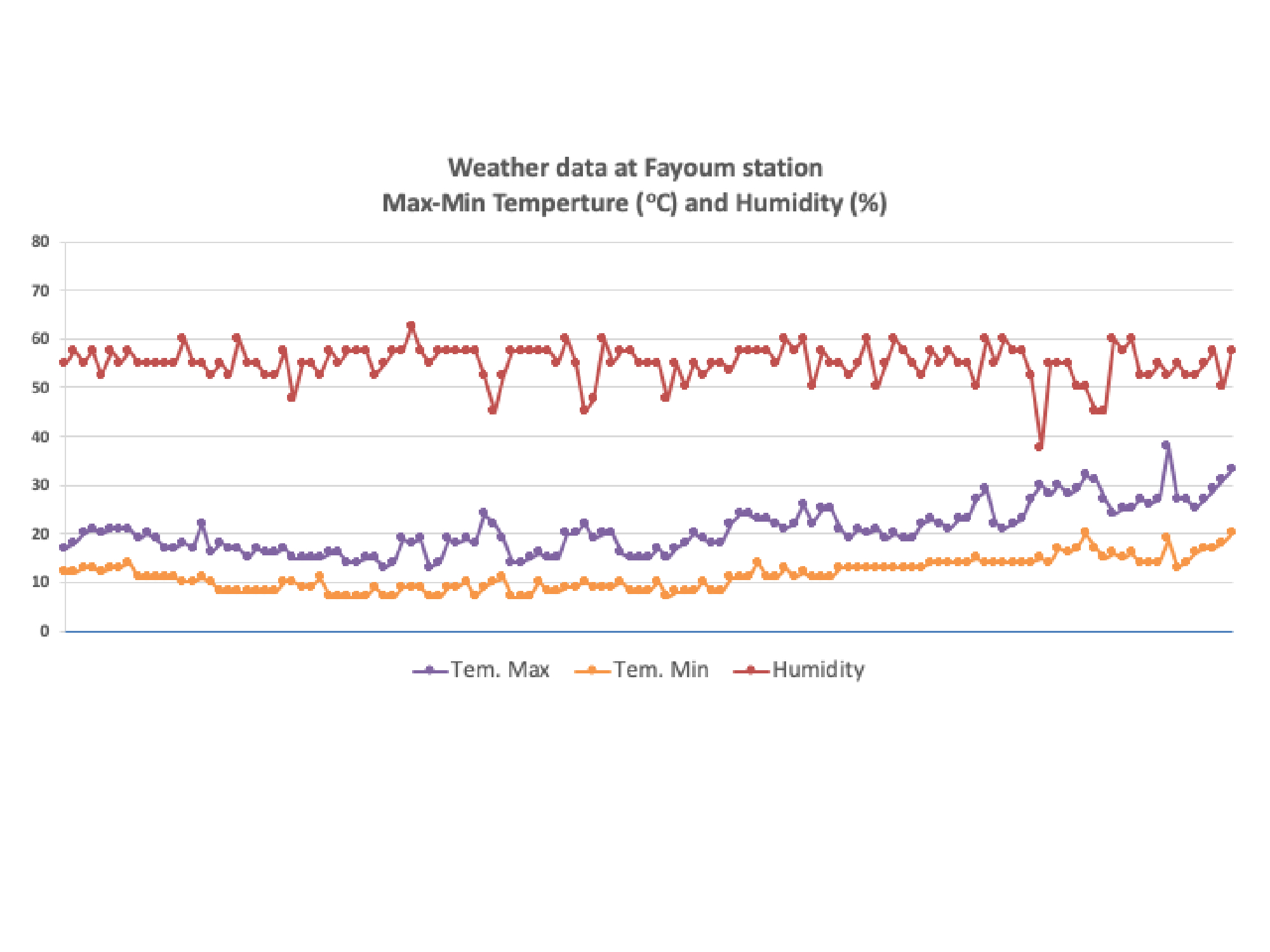

Supplement: Supplementary file 1 [file genes-11-00533-s001.zip › genes-765865-supplementary/Figure S2.jpg]

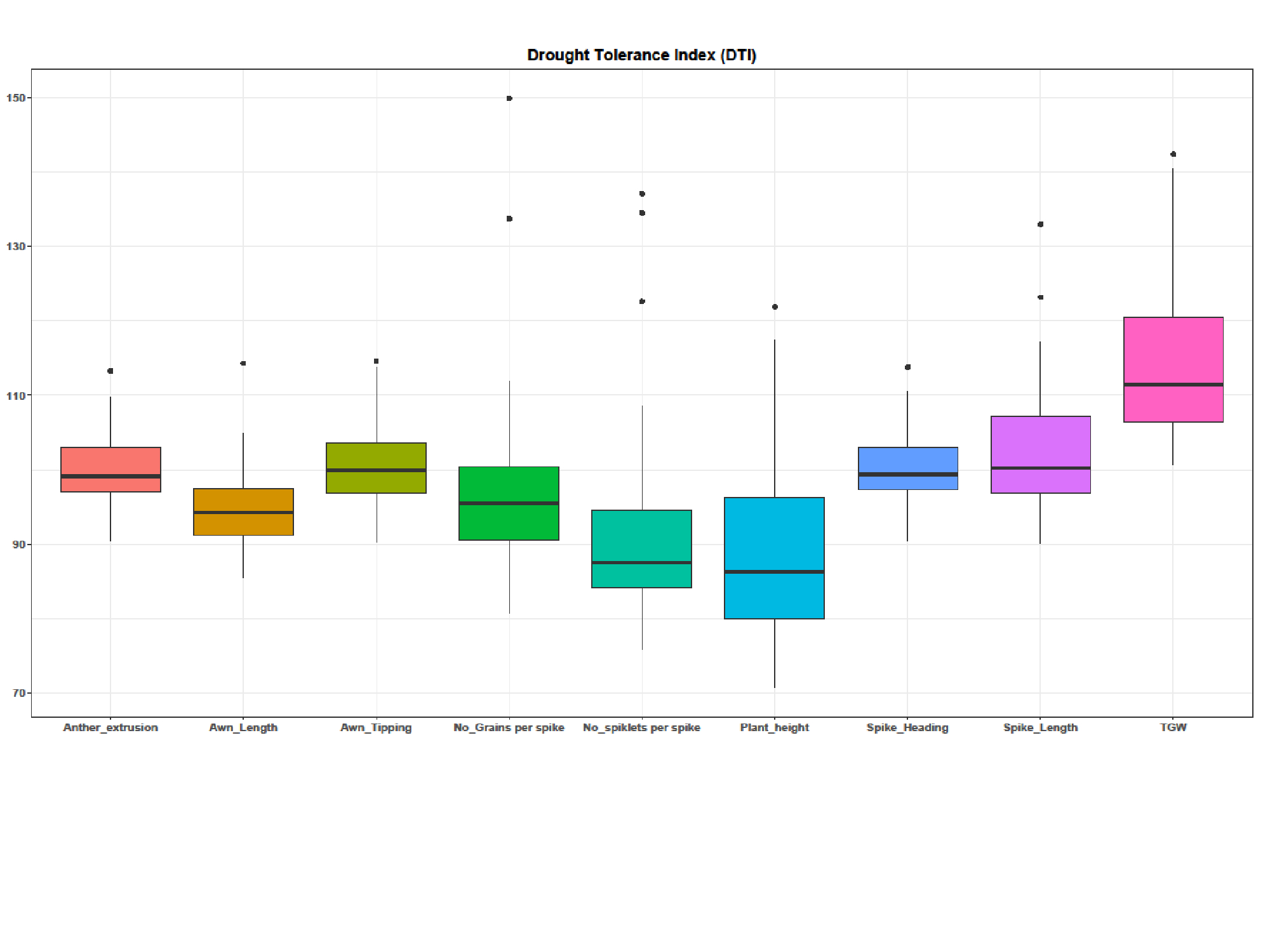

Supplement: Supplementary file 1 [file genes-11-00533-s001.zip › genes-765865-supplementary/Figure S4.jpg]

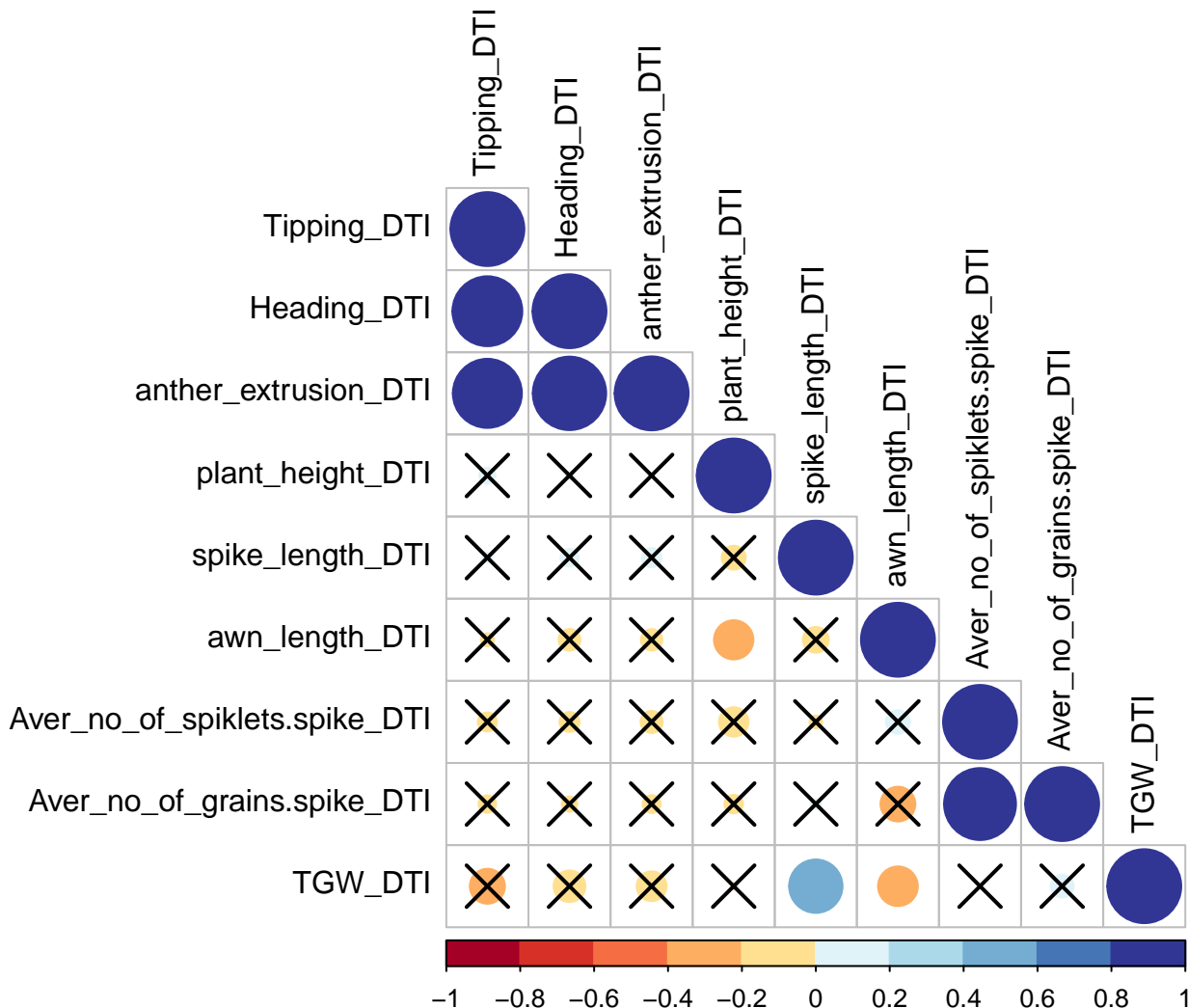

Supplement: Supplementary file 1 [file genes-11-00533-s001.zip › genes-765865-supplementary/Figure S5.pdf]
